# Assessing community variation and randomness in public health indicators

**DOI:** 10.1186/1478-7954-9-3

**Published:** 2011-02-02

**Authors:** Stephan Arndt, Laura Acion, Kristin Caspers, Ousmane Diallo

**Affiliations:** 1 Department of Psychiatry, Carver College of Medicine, University of Iowa, Iowa City, Iowa 52242 USA; 2 Department of Biostatistics, College of Public Health, University of Iowa, Iowa City, Iowa 52242, USA; 3 Iowa Consortium for Substance Abuse Research and Evaluation, 100 Oakdale Campus M308 OH, University of Iowa, Iowa City, Iowa 52245-5000, USA; 4 Department of Epidemiology, College of Public Health, University of Iowa, Iowa City, Iowa 52242, USA; 5 Iowa Department of Public Health, Des Moines, Iowa, USA; 6 Department of Occupational & Environmental Health, College of Public Health, University of Iowa, Iowa City, Iowa 52242, USA

## Abstract

**Background:**

Evidence-based health indicators are vital to needs-based programming and epidemiological planning. Agencies frequently make programming funds available to local jurisdictions based on need. The use of objective indicators to determine need is attractive but assumes that selection of communities with the highest indicators reflects something other than random variability from sampling error.

**Methods:**

The authors compare the statistical performance of two heterogeneity measures applied to community differences that provide tests for randomness and measures of the percentage of true community variation, as well as estimates of the true variation. One measure comes from the meta-analysis literature and the other from the simple Pearson chi-square statistic. Simulations of populations and an example using real data are provided.

**Results:**

The measure based on the simple chi-square statistic seems superior, offering better protection against Type I errors and providing more accurate estimates of the true community variance.

**Conclusions:**

The heterogeneity measure based on Pearson's χ^2 ^should be used to assess indices. Methods for improving poor indices are discussed.

## Background

Evidence-based health indicators are vital to needs-based or results-based programming. Agencies frequently make programming resources available to local jurisdictions based on need. In 2008, the United States Department of Health and Human Services distributed more than $421 million in Mental Health Block Grant funds based, in part, on the number of people at risk within each state [1]. Each state then disperses funds to local communities. The amount dispersed is often determined by a demonstrable index of need.

The indicators used in public health funding contexts vary considerably. Common indices include census counts within a certain age group or the percentage of people reporting a particular behavior from a population-based surveillance survey, e.g., the percentage of people reporting binge drinking in the past 30 days. Mortality, arrest, remission, or recidivism rates are also commonly used by different funding agencies. US government agencies such as the Centers for Disease Control and Prevention provide yearly datasets such as the Behavioral Risk Factor Surveillance System (BRFSS) that include prevalence and trend data. State governments and other agencies support various other surveillance systems for local assessments. For example, the state of Iowa supports the administration of the Iowa Youth Survey to all 6^th^, 8^th^, and 11^th ^graders in the state every three years.

The use of objective indicators in making funding decisions can be very attractive for policymakers and funders. A simple formula to determine which community receives programming funding is transparent and appears unbiased [2,3]. Targeting areas with high need also appears to be a rational and evidence-based approach. In the United States, there has been a recent effort to rank the health of counties within states using a collection of indicators [4,5]. Rankings or "league tables" are extremely intuitive and make identification of those locales with the greatest need deceptively easy. However, this effort relies on two very basic assumptions - that the communities differ and that communities with the highest (or lowest) indicators truly reflect the communities with the greatest need for public health funding [6].

Similar issues arise in the pay-for-performance programs that private health insurers, Medicare, and Medicaid use in the US and that the National Health Service uses in United Kingdom. Pay-for-performance necessarily requires using indices, often outcome indicators, for rewards. Whether ranking hospitals or other institutions or regions, the same assumption is made - that the ranking indicators mostly reflect performance rather than error.

An indicator would show a poor connection with community needs or outcomes if the differences among communities mainly reflected random variation. For example, the BRFSS estimates of the percentage of adults who drink heavily are based on a sample. Other nonsurvey-based data are incomplete as well - for example, outcomes of random compliance checks for liquor or tobacco sales to minors. Whenever there is a possible sampling error, observed differences among communities may be at least partially dictated by random error. The question for policymakers is: How much of the diversity among communities is error, and how much is real variation?

While this question might be answered by reviewing the communities' indicator estimates and their standard errors, this quickly becomes daunting. With more than a few communities or more than two indices, a summary statement quantifying each indicator would be invaluable in deciding their relative worth. Aside from lack of convenience, there are other problems with simply relying on the standard errors [6]. One basic issue is that the standard error of the indicator is not the standard error of the ranking, i.e., knowing the accuracy of a single measurement does not indicate the accuracy of that estimate's ordering relative to the other communities. The relationship of standard errors of the individual estimates to the standard errors of the relative rankings is complex [7-9]. From another venue of biostatistics, Gauch has noted that the problems of ranking and selection are different statistical questions than accuracy of estimation [10,11]. For example, the community with the highest estimated rate of obesity might have a relatively large standard error, but that rate may still be substantially distinguishable from the community with the next highest rate. Conversely, the standard errors might be very small, but the communities might be very homogeneous, making the resolution difficult.

From a technical perspective, the statistical literature provides a more formalized treatment. A region's rank can be formalized as the number of times a particular region, say Region d, has a rate (*p*_d_) that is larger than other regions, e.g., ∑(*p*_d _≥ *p*_d'_), for all regions d and d' and where the value within the parentheses equals 1 when it is true and 0 otherwise. Thus, each region is compared to all regions, for a total of k^2 ^comparisons, where k is the total number of regions. Of course, because rates within any region are measured with some amount of error, there is a degree of uncertainty regarding any comparison (*p*_d _≥ *p*_d'_). One suggestion from the small area estimation literature is to replace the ranks with the sum of the estimated probabilities for each estimated rate, i.e., ∑[prob(p^d≥p^d')], where the simple rates are replaced by the small area estimates [12,13]. This method tends to "shrink" the ranks toward the median as a function of the spread of the estimates as well as the size of the estimated standard errors. Each of the possible comparisons also has a covariance that needs to be considered, again magnifying the complexity of the problem. Furthermore, the resulting sums of the probabilities are not actually ranks, making interpretation difficult. Rao, in his seminal work on small area estimation, suggests using triple goal estimators of Shen and Louis when performing Bayesian estimates for regional values [14,15]. Of particular interest is that the triple goal method explicitly includes the rank ordering and adequate interregional spread in the loss functions used by the Bayes estimates. More importantly, the triple goal method explicitly attempts to provide good estimates of the relative regional ordering rather than simply good estimators for the rates, goals that are not completely overlapping and will not necessarily result in the same estimates.

In actual application, two studies of health indicator performance (mortality rates and lead poisoning) across a variety of geographic levels noted that the degree of community homogeneity affected how well the indices performed [16,17]. The degree of community homogeneity is not necessarily related to the size of the local population or the corresponding size of the standard error or estimate. In the context of hospital rankings on performance measures, one English study noticed considerable variation in the rankings, as much as half of the league table [18]. A similar result regarding the instability of rankings is given by O'Brien and Peterson regarding hospital mortality rankings [19]. These considerations may also explain inconsistencies in health rankings using different indicators across provinces [20] and communities [21] in Canada.

Both between-community heterogeneity and within-community homogeneity must be considered simultaneously when assessing an index's performance in rankings. The present paper offers two proposed methods for assessing this issue, considering both within- and between-community homogeneity simultaneously.

We compare the statistical performance of two heterogeneity measures applied to community differences on a surveyed index. These measures may be useful to screen indicators for heterogeneity among communities due to true differences versus sampling error. The measures, ideally, would be useful for policymakers to choose appropriate indicators for resource allocation, funding, performance pay, ranking, and reporting. These heterogeneity measures would not correct indicators for sampling variability, but they would identify those indicators showing more random variation or noise.

The two measures, one based on the work of DerSimonian and Laird (DL) [22] and one based on a simple Pearson's (P) chi-square, $I_{DL}^{2}$ and $I_{P}^{2}$, assess the degree to which the variation among communities corresponds to the variation expected by chance (*I*^2 ^≤ 0), or if the variation exceeds that caused by chance (*I*^2 ^→ 1). Both of the *I*^2 ^measures have associated statistical tests to determine if the variation is significantly different from chance expectations. Both measures also provide a means to estimate the actual range of real differences. For this application, we restrict the discussion to indicators or outcomes that are simple proportions, e.g., the percentage of people who report heavy drinking within the last 30 days.

### Statistical background

A measure of heterogeneity or differences among units, $I_{DL}^{2}$, was recently suggested in a meta-analysis context [23,24]. The main goal of meta-analysis is to combine results from a variety of studies on a topic and to summarize and quantify the combined outcome. In meta-analyses of clinical trials, *k *independent studies report a treatment effect and its standard error. For the current application, independent communities report an incidence or prevalence, *p_i_*, where *i *reflects the *i*^th ^community. In a meta-analysis, the presence of heterogeneity is a nuisance that requires specialized statistical treatment. However, in our context, the heterogeneity measure is the item of interest.

The $I_{DL}^{2}$ measure used in meta-analysis is based on Cochran's Q, [25,26] as modified by DerSimonian and Laird, [22]*Q_DL_*. First, *Q_DL _*is calculated; next, *Q_DL _*is converted to $I_{DL}^{2}$; lastly, the variation among communities can be estimated. The *Q *statistic, used to test for heterogeneity, is distributed as χ^2 ^with *k *- 1 degrees of freedom (df). Under the null hypothesis that the studies are homogeneous, the expected value of *Q_DL _*(i.e., a χ^2^) equals the df. Thus, *Q_DL _*is a test that $I_{DL}^{2}$ differs from zero.

Only a little modification to the meta-analysis notation is necessary to fit *Q_DL _*and $I_{DL}^{2}$ to the present situation. Weights (w_i_) used for the calculation of *Q_DL _*are based on the inverse of the sampling variance ($s_{i}^{2}$) within a community rather than a study. When the outcome measure is binary and estimated from independent observations, the sampling variance for the i^th ^community, $s_{i}^{2}$, is *p*_*i*_(1 - *p*_*i*_)/*n*_*i*_, where *n_i _*is the total number of observations within the community and *p_i _*is the proportion of positive cases. Following DerSimonian and Laird [22], we use the weights, $w_{i}=1/s_{i}^{2}$, to create a pooled estimate across all units, *p*_0 _= Σ*w*_*i*_*p*_*i*_/Σ*w*_*i*_. The test statistic is, *Q*_*DL *_=Σ*w*_*i*_(*p*_*i *_- *p*_0_)^2^.

The following equation converts *Q_DL _*to $I_{DL}^{2}:I_{DL}^{2}=(Q_{DL}-df)/Q_{DL}$. The *Q_DL _*value represents a standardized measure of the observed variance among the *k *communities, and *Q_DL _*minus the df value represents the degree of variance among communities that exceeds the chance expectation. Thus, $I_{DL}^{2}$ indicates the proportion of true community heterogeneity over the total observed variation. An $I_{DL}^{2}$ of 0.75 would suggest that 75% of the variation is not error variation in need or outcome among the communities. This interpretation of *I*^2 ^led several investigators to point out the resemblance of $I_{DL}^{2}$ to an intraclass correlation coefficient used to assess the reliability [23,27,28]. As noted by Shrout and Fleiss, the intraclass correlation can, under certain conditions, be negative [28]. Similarly, $I_{DL}^{2}$ can be less than zero if the observed variation is less than expected. In practice, values less than zero are reported as zero.

Finally, DerSimonian and Laird [22] show that the true (nonerror) between-community variation can be estimated using: $s_{communities}^{2}=\max\{0,(Q_{DL}-df)/[\Sigma w_{i}-(\Sigma w_{i}^{2}/\Sigma w_{i})]\}$. The numerator contrasts the observed *Q*-value minus its expectation (i.e., the df), which reflects the degree that the observed *Q*-statistic exceeds the random noise. The denominator returns *Q *to the original metric. Thus, this value is interpretable as the actual variation among the units, existing beyond random noise, and in the original units, the incidence rates.

As an alternative to the DL method, we also include a method based on a simple Pearson's χ^2 ^statistic. For example, the *k *communities would represent the rows of a two-way frequency table, the two column entries would represent the number of people reporting or not reporting a behavior, and the χ^2^-statistic can be calculated in the usual way. An algebraically equivalent form of the χ^2^-statistic is Σ*w*_*i*_(*p*_*i *_- *p*_•_)^2^, where *w*_i _is the inverse of that community's squared standard error $s_{i}^{2}$, but now *p*_• _is the overall (marginal) incidence rate across all communities [29].

Replacing Q in the formula for $I_{DL}^{2}$ with Pearson's χ^2 ^gives, $I_{P}^{2}=(\chi^{2}-df)/\chi^{2}$. Since $I_{P}^{2}$ represents the proportion of variance among communities that exceeds the random noise due to sampling error, and this measure is analogous to the intraclass correlation, i.e., $s_{communities}^{2}/(s_{communities}^{2}+s_{error}^{2})$, the actual variance among communities should be approximately $I_{P}^{2}\times s_{observed}^{2}$, where $s_{observed}^{2}$ is the calculated variance among community rates.

## Methods

### Sources of data

Data from the BRFSS, which involves yearly telephone interviews across the nation, were used to illustrate these methods. This is a complex, multistage survey. However, for this application, we only analyzed raw numbers from 2007 and 2008. The actual county-level data we used were summary 30-day prevalence rates for binge drinking for the 99 counties in Iowa, data available at the county level from the Iowa Department of Public Health.

The other source of illustrative data came from summary reports of the 2008 administration of the Iowa Youth Survey (IYS) [30]. The IYS is Web-based, in-school survey of all 6^th^, 8^th^, and 11^th ^graders in public and private schools administered by the Iowa Department of Public Health. Coverage is 83.5% of the enrolled student population in Iowa, and 97,741 validated records were received from students across the state.

Simulation studies. Simulations were performed within MATLAB. MATLAB calculations for $I_{DL}^{2}$ and the modified *Q_DL _*[22] were validated against the Stata implementation of these statistics [31].

To assess the Type I error rates for *Q_DL _*and Pearson's χ^2 ^within this context, we randomly generated *k *independent binomial proportions representing *k *communities, each with 200 observations. We chose two proportions in the simulations: 0.5 and 0.1 to represent relatively common and less common rates. During each simulation, the fixed population proportion (0.5 or 0.1) would yield samples (n_i _= 200) with observed proportions varying solely due to sampling error. Simulations generated results for differing numbers of communities (*k*): 20, 30, 50, and 100. We also used varying numbers of observations within each simulation (ranging from 100 to 300). Because these results did not substantially differ from those using the fixed sample size of 200, only the fixed sample sizes are shown. We were interested in the Type I error rate using a nominal alpha level of 0.05. Using 20,000 replications for each simulation provides an (asymptotic) Monte Carlo error [32] of 0.0015, with the exact confidence interval of 0.0470 - 0.0531.

Another simulation assessed the resemblance of the two *I*^2 ^measures and the intraclass correlation. For each of 20,000 iterations, we randomly selected a range of incidence rates among communities using a uniform distribution. The population incidence among communities ranged from a single fixed value of 0.5 to 0.5 ± 0.4; thus, the spread among communities would be 0 (center = 0.5, variance = 0) to 0.8 (center = 0.5, range 0.1 - 0.9, variance = 0.0533). Individual community rates were randomly selected within the span and defined a community's true rate. Using that rate, we generated two independent samples. This mimics a situation where the set of communities was sampled two separate times. The two samples from the same population parameters allowed us to calculate an estimated intraclass correlation. The values of $I_{DL}^{2}$, $I_{P}^{2}$, and the observed variance among the communities were recorded.

## Results

Table 1 shows the observed Type I error rates for *Q_DL _*and the simple Pearson's χ^2 ^-test. After each of 20,000 replications, we noted whether the table value for *Q_DL _*or χ^2 ^was "significant" at the nominal level of 0.05. The χ^2^-test consistently showed an observed Type I error rate close to 0.05. In no instance did the observed Type I error rate go outside the 95% confidence interval (0.0470 - 0.0531) for this number of replications. However, the *Q_DL_*-test consistently signaled heterogeneity too often, with every Type I error rate exceeding the upper limit of the confidence interval. The problem with the *Q_DL_*-test seems to increase with increasing numbers of communities. This is particularly apparent in the case of lower population incidence rates. Here, the observed Type I error rate clearly exceeds any reasonable expectation, even for an approximation, and errantly overidentifies heterogeneity.

**Table 1:** Type I error rates (P) with 20,000 simulations and 200 observations per community

	Population Incidence Rate			
	
	0.5		0.10	
**Number of Communities**	***Q***_***DL***_	**Pearson's χ**^**2**^	***Q***_***DL***_	**Pearson's χ**^**2**^

20	0.0602	0.0525	0.0836	0.0494
30	0.0601	0.0496	0.0917	0.0498
50	0.0619	0.0496	0.1060	0.0489
100	0.0642	0.0485	0.1295	0.0502

Note: 95% confidence bounds for P = 0.05 are 0.0470, 0.0531

**Figure 1:**
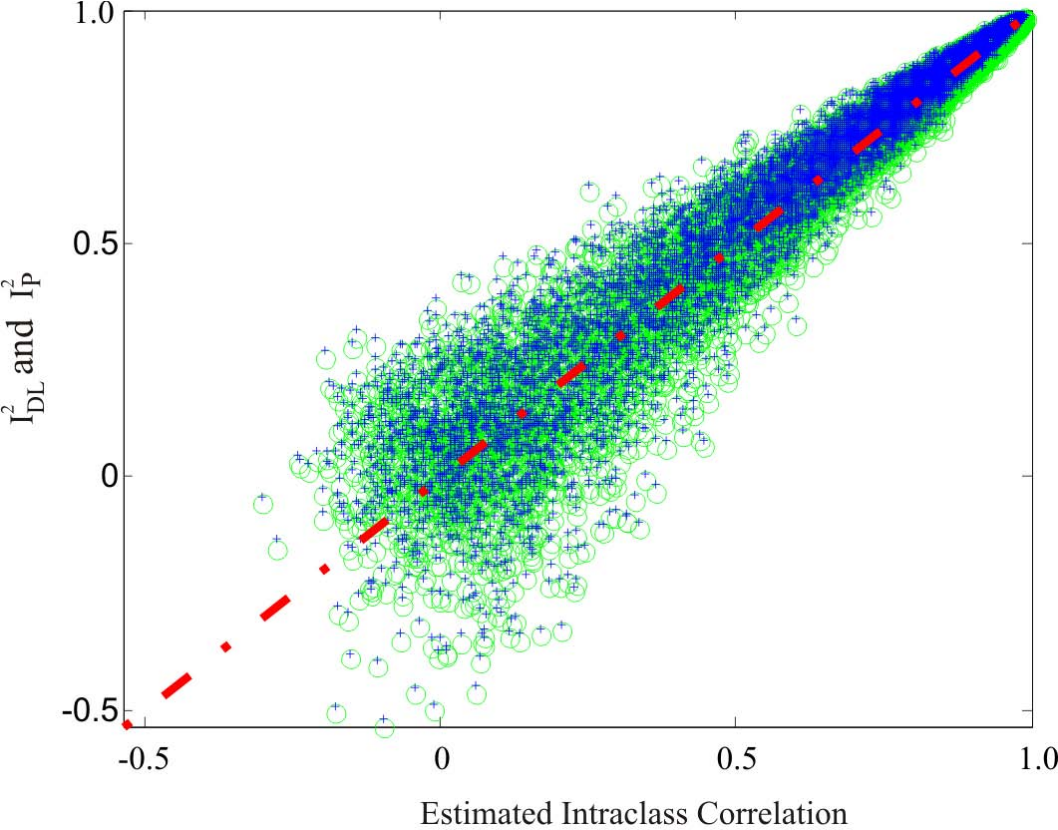
**Plot of intraclass correlations and $I_{DL}^{2}$ (blue +) and $I_{P}^{2}$ (green o) values against 100% agreement (red line)**.

In the second simulation, the spread among communities was varied randomly from 0 to 0.8, centered at 0.5, which corresponds to between-community variances of 0 to 0.0533. Two samples were taken from each of 20,000 randomly sampled populations in order to calculate interclass correlations estimating the proportion of between-community variation to total variation (between and within). Figure 1 shows the plot of the intraclass correlations versus $I_{DL}^{2}$ (blue +) and $I_{P}^{2}$ (green o). The Pearson correlations between the intraclass correlation and both $I_{DL}^{2}$ and $I_{P}^{2}$ were all greater than 0.98. Similarly, the correlations between $I_{DL}^{2}$ and $I_{P}^{2}$ was 0.97. The Spearman correlations were all greater than 0.99.

Thus, $I_{DL}^{2}$ and $I_{P}^{2}$ both measure a construct similar to the intraclass correlation. One subtle difference between $I_{DL}^{2}$ and $I_{P}^{2}$ is visible in the figure. $I_{DL}^{2}$ produces slightly but consistently larger estimates than $I_{P}^{2}$. For the first of the pair of values from each population, the difference averages 0.0112 (SD = 0.0024) with a range of 0.0076 to 0.0382. This difference increases with lower intercommunity variation (Spearman r > -0.68). The larger values for $I_{DL}^{2}$, particularly for low real values, corresponds to the increased Type I error rate with this measure.

**Figure 2:**
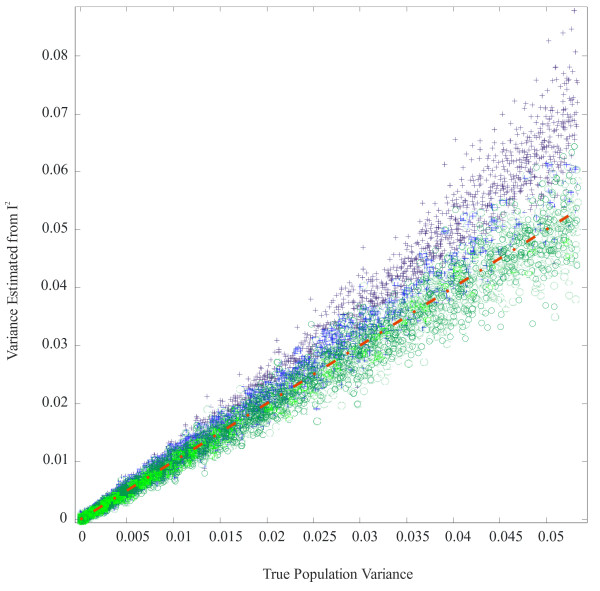
**$I_{DL}^{2}$ (blue +) and $I_{P}^{2}$ (green o) estimated and actual variances (red line) in 20,000 simulated samples**.

In a final set of simulations, we used $I_{DL}^{2}$ and $I_{P}^{2}$ to estimate the real variation among communities. While calculations were performed on 20,000 replicated samples, Figure 2 shows the results for 5,000 randomly sampled estimates based on $I_{DL}^{2}$ (blue +) and $I_{P}^{2}$ (green o). In these simulations, the population with the largest possible variance produced communities ranging from an incidence of 0.1 to 0.9 (range = 0.8, variance = 0.0533). The red line in Figure 2 shows the true variance among the communities (excluding sampling error). Clearly, the estimates based on $I_{DL}^{2}$ were nearly always too large and very frequently exceeded the possible limit.

Simulations were also performed where the intercommunity variance was preset to 0 (no community variance), 0.02, 0.04, and 0.06. The overall incidence rate was 0.5 in 100 communities, with 200 observations per community. A variance of 0.02 corresponds to the communities ranging 0.5 ± 0.245 (i.e., ±0.5 * sqrt[variance * 12]) with a uniform random distribution. These estimates are shown in Table 2.

**Table 2:** Mean (SE) Estimated Community Variance based on $I_{DL}^{2}$ and $I_{P}^{2}$, based on 20,000 random samples

		Estimate Based on:	
**Community Variance**	**Observed Variance**	$I_{DL}^{2}$	$I_{P}^{2}$

0	0.000	0.000	0.000
	(0.000)	(<0.001)	(<0.001)
0.02	0.020	0.022	0.020
	(0.002)	(0.002)	(0.002)
0.04	0.040	0.048	0.039
	(0.004)	(0.005)	(0.004)
0.06	0.060	0.083	0.059
	(0.005)	(0.008)	(0.006)

Note: Population incidence rate was 0.5 for all replications. Communities varied using a uniform distribution.

Here again, the measure $I_{DL}^{2}$ overestimates the actual nonerror variance among communities when there actually is variation, and the overestimation increases with greater variance. The $I_{P}^{2}$ measure appears to reconstruct the variance accurately. Another set of simulations (not shown) used a population incidence rate of 0.15 and with variances from 0 to 0.00005, with the same pattern of results.

### Binge drinking in Iowa counties

Across the 99 counties in Iowa, there were 8,301 individuals who responded to BRFSS survey questions in 2007 and 2008 regarding binge drinking during the past 30 days. Statewide, 1,268 people responded positively, and 7,033 responded negatively. The counties varied widely in both sample size and the 30-day incidence rates. The mean sample size within counties was 85.59 (SD = 143.95), and the median was 44 (range: 15 - 1,136), reflecting the large number of rural counties in the state. The mean 30-day prevalence was 0.145 (SD = 0.06), which ranged from a low of 0 to a high of .334.

Compared to the BRFSS, the Iowa Youth Survey data included a much larger number of responses from Iowa youth, with a mean sample size of 971. The mean county rate for binge drinking was 0.1324 (SD = 0.034), ranging from 0.678 to 0.251.

Analysis results of the community variation appear in Table 3. One of the smaller counties in Iowa only included 17 BRFSS interviews, of which none reported binge drinking. This caused problems for calculating $I_{DL}^{2}$. The within-county variance was zero, and this appears in a denominator, resulting in an undefined number.

**Table 3:** Community variation analysis using the BRFSS and IYS for 30-day binge drinking

	BRFSS	**BRFSS**^**1**^	IYS
Df	98	97	98
*Q_DL_*	-	154.13	660.54
Probability	-	0.0002	<0.0001
$I_{DL}^{2}$	-	0.371	0.852
Estimated $s_{i}^{2}$	-	0.00084	0.00064

χ^2^	137.42	134.12	691.31
Probability	0.0053	0.0075	<0.0001
$I_{P}^{2}$	0.287	0.277	0.858
Estimated $s_{i}^{2}$	0.00061	0.00058	0.00067

^1^This column excludes one small county with a zero incidence.

Both test statistics, *Q_DL _*and Pearson's χ^2^, agree that the communities vary more than by chance, suggesting actual heterogeneity. Interestingly, the rank ordering of Iowa counties using these two different data sources shows significant, but not strong, agreement, Spearman r = 0.21 (95% confidence interval [CI]: 0.01, 0.39). This also suggests that some communities exhibit higher rates of binge drinking than others. The low value of the correlation is influenced in part by the degree of error, particularly in the BRFSS estimates.

The two measures assessing the degree of true community variation, $I_{DL}^{2}$ and $I_{P}^{2}$, disagree somewhat using the BRFSS data, although both suggest that less than 40% of the community variation is attributable to actual community differences. By implication, more than 60% of the community differences are attributable to chance. The mean BRFSS sample size of a little more than 85 interviews per community is, at least in part, responsible for the high degree of error. With that mean sample size and a true population prevalence of 0.1412, the exact confidence interval ranges from 0.075 to 0.234.

The IYS data represent a much larger sample size. Both heterogeneity measures show close agreement for the IYS data and suggest that this is a fairly strong measure, with more than 85% of the community variation due to community differences. Interestingly, the estimates of the actual community variance for the BRFSS and IYS are in close agreement, variance roughly 0.0006, based on the simple χ^2 ^procedure. Naively using a uniform distribution, this corresponds to a true range of incidence rates of 8.49% centered near a 15% binge drinking rate, i.e., 15% ± 4.24. While the estimates of the range of incidence rates are similar, the Spearman correlation of only 0.21 suggests that the county binge drinking rankings are not entirely similar. Indeed, looking at the raw data and selecting the 10 highest binge-drinking counties according to the BRFSS, only one county was so ranked according to the IYS.

## Discussion

We presented three different uses for the homogeneity measures, testing whether or not there are real differences among communities, measuring the degree of actual heterogeneity using *I*^2^, and estimating the actual amount of variation among rates. Since only values of *I*^2 ^that are significant suggest any real heterogeneity, our results suggest the first step would be to use Pearson's χ^2 ^test. Providing there is evidence for real heterogeneity so that there is reason to believe that *I*^2 ^is greater than zero, the next step involves estimating $I_{P}^{2}$ and perhaps the actual variance.

The statistical test is important but only as a gatekeeper and antecedent. When there are many communities and large sample sizes, trivial heterogeneity may be significant. With 50 communities, an *I*^2 ^value of 0.36 will be statistically significant (P < 0.05), even though that value of *I*^2 ^may be unimpressive for practical considerations. When evaluating indices for policy decisions, only those demonstrating *I*^2 ^values closer to 1.0 will be of interest.

Interpretation of these heterogeneity measures is, in one sense, straightforward. Since *I*^2 ^bears a resemblance to the intraclass correlation, 100 × *I*^2 ^can be interpreted as the percentage of the variability among communities that is due to real differences among them. The complement, 1 -*I*^2^, is the proportion of error in the index. The magnitude for what constitutes a good measure based on $I_{P}^{2}$ will vary depending on the situation; however, values greater than 0.75 to 0.80 might be minimal for decision-making. A value of 0.8 implies that 80% of the variability among communities reflects real differences, while error accounts for only 20%. $I_{P}^{2}$ values closer to 0.5 might be mildly suggestive.

In our illustrative data, the IYS showed good measurement quality, with *I*^2 ^values greater than 0.85. Community variability on this index was 85% real and 15% random noise. The county differences in the BRFSS binge-drinking indicator were mostly random variability, with *I*^2 ^values less than 0.40, i.e., 40% actual variability and 60% error. Lower values of *I*^2 ^suggest that the indicator should be enriched or supplemented. Increasing the sample size would enrich the indicator by reducing the amount of noise for the community estimates. While it may not be practical to increase sample size for the BRFSS, the same end might be achieved by considering multiple years of data. Alternatively, supplementing the indicator with additional correlated indicators may produce a more acceptable composite summary indicator. Thus, we tentatively suggest that stand-alone indices should have *I*^2 ^values near or above 0.8 for policy decisions. Lower-valued indices may still be useful but would likely need to be supplemented with other indices or information. Another interesting suggestion would be to weight composite indicators as a function of their *I*^2 ^values.

Of course, particularly poor performance of an indicator may suggest that there is little or no community variation in the trait of interest. Even when significant, the estimated intercommunity variance, $s_{i}^{2}$, gives an actual suggestion of how big the differences are in terms of the raw rates. With a large enough sample size for many communities, an indicator may provide a high *I*^2 ^value, but the actual variation may be epidemiologically or clinically trivial.

We contrasted two different methods, one based on meta-analysis using DerSimonian and Laird's work [22] and one based on a simple Pearson's χ^2^. From a purely statistical perspective in this context, the performance of the simple Pearson's χ^2 ^was superior to the DL method. The Pearson χ^2 ^method is easy to calculate and offers better protection for Type I errors. $I_{P}^{2}$ tends to mirror the intraclass correlation better and provides more accurate estimates of the true community variation in rates when compared to $I_{DL}^{2}$. The calculation of $I_{DL}^{2}$ also becomes undefined if any of the community rates is zero. Thus, the Pearson-based method has much in its favor.

Zero counts in communities preclude calculation of $I_{DL}^{2}$ and may cause issues for $I_{P}^{2}$, especially if there are more than a few such counts. Most assessments of the adequacy of Pearson's χ^2 ^in sparse tables are in the context of smaller cross-tabulation tables. Even then, these assessments focus on how well the χ^2 ^approximation provides adequate probability estimates for a hypothesis test [29]. The purpose of the χ^2 ^estimate used here is very different since it is the basis of $I_{P}^{2}$. Furthermore, the number of communities involved tends to produce a table with a larger number of rows than is typical of a cross-tabulation table in most analytical applications. One early paper suggests that χ^2 ^may still function adequately with low frequencies of observations, although perhaps with a correction (i.e., using df = k - 2 instead of k - 1) [33]. More work may be required to adequately assess how well $I_{P}^{2}$ functions with zero counts, and it may possibly need to be adjusted using two stage models [34], mixture models [35], or a generalized Poisson distribution [36].

The DL technique readily applies to health indices other than rates. Means of behaviors (e.g., number of drinks, miles driven) or other indices are appropriate provided standard errors are available. For example, many national datasets use complex sampling procedures producing data where many basic assumptions (e.g., independence) are violated. These data require Taylor series or other approximations to produce the standard errors around means, rates, or quintiles [37]. In these more complex situations, the estimated standard errors provide the information required to produce the weights needed for calculating the DL-based method. However, the generalization of the Pearson method is still lacking, and we have only assessed the performance of these methods when using rates and percentages in this paper.

Other measures of heterogeneity exist, and we only evaluated two. In part, our decision was based on these measures' ease of interpretation. For example, Higgins and Thompson introduced their statistic, *H*. Like $I_{DL}^{2}$, *H *is based on the *Q*-statistic; however, it cannot be interpreted as a percentage of variance due to heterogeneity. Another study of heterogeneity measures introduces a measure similar to $I_{P}^{2}$, but it is based on *Q *rather than Pearson's χ^2 ^[38]. Sidik and Jonkman [39] recently evaluated seven variants of heterogeneity measures. Further study is clearly needed to assess these alternatives in the current context.

The heterogeneity measures also have some limitations. Both the DL and Pearson methods are large sample approximations; however, for most epidemiological applications, this will not pose problems since the sample size requirements are fairly low, e.g., expected values greater than 5 in 80% or more of the communities [29]. Power has been cited as a problem with the Q-test, but this is more of an issue for meta-analyses of clinical trials where the number of studies (here, communities) and the number of subjects is small in terms of typical epidemiological surveillance standards [40]. For example, with a true value for *I*^2 ^= 0.5 and based on a power analysis for the Pearson's χ^2^-test, there is more than 89% power to detect it in as few as 10 communities. One limitation of our study is that we used a sample size of 200 per community for our simulations. We also performed simulations where we allowed the sample sizes to vary (from 100 to 300). This corresponds to between one and two years of BRFSS data for US counties. Thus, our results may not generalize to indices measured on fewer numbers of observations. Furthermore, we did not assess the adequacy of either of the *I*^2 ^measures when sample sizes might be grossly imbalanced. Finally, these measures of heterogeneity and their significance tests assume independent observations. In this context, spatial or geographic correlations among the communities would violate this assumption. Semi-variograms of the exemplar data used here did not demonstrate noticeable spatial correlation; however, such checks should be performed before using these methods.

## Conclusions

When using indicators to decide how to target health resources, the indicator should be assessed for its ability to reflect true underlying community differences. Actual variation in health needs, rather than chance variations, should guide decisions about programming and resource allocation. $I_{P}^{2}$ showed good statistical qualities and is suggested as an assessment tool for determining the quality of health indicators.

## Competing interests

The authors declare that they have no competing interests.

## Authors' contributions

SA conceived the idea, performed analyses, and wrote the first draft. LA worked on and reviewed the statistical development and contributed to the writing. KC reviewed the statistical development and contributed to the writing. OD provided data and reviewed the development and writing. All authors read and approved the final manuscript.
